# Health problems among family caregivers of former intensive care unit (ICU) patients: an interview study

**DOI:** 10.3399/bjgpopen20X101061

**Published:** 2020-08-26

**Authors:** Dries van Sleeuwen, Floris van de Laar, Wytske Geense, Mark van den Boogaard, Marieke Zegers

**Affiliations:** 1 Department of Intensive Care Medicine, Department of Primary and Community Care, Radboud University Medical Center, Nijmegen, The Netherlands; 2 Department of Primary and Community Care, Radboud University Nijmegen Medical Center, Nijmegen, The Netherlands; 3 Department of Intensive Care Medicine, Radboud Institute for Health Science, Radboud University Medical Center, Nijmegen, The Netherlands; 4 Department of Intensive Care Medicine, Radboud Institute for Health Sciences, Radboud University Medical Center, Nijmegen, The Netherlands; 5 Department of Intensive Care Medicine, Radboud Institute for Health Sciences, Radboud University Medical Center, Nijmegen, The Netherlands

**Keywords:** caregivers, critical illness, general practice, intensive care, critical care, mental health, qualitative research, primary health care

## Abstract

**Background:**

Family caregivers of former intensive care unit (ICU) patients may suffer from physical and mental problems owing to ICU hospitalisation of their loved ones. These problems can have a major impact on their daily lives. Little is known about experienced consequences of ICU hospitalisation on caregivers in general practice.

**Aim:**

To explore health problems in family caregivers of former ICU patients and the consequences in their daily lives.

**Design & setting:**

Semi-structured interviews with family caregivers of former critically ill patients treated in a Dutch ICU.

**Method:**

Purposively sampled relatives of former ICU patients were interviewed between April and May 2019. Interviews were conducted until data saturation was reached. Interviews were then thematically analysed.

**Results:**

In total, 13 family caregivers were interviewed. The interviews took place 3 months to 3 years after ICU discharge. Expressed problems were categorised into six themes: (1) physical functioning (for example, tiredness, headache, and feeling sick more often); (2) mental health (for example, anxiety, more stress and difficulty in expressing emotions); (3) existential dimension and future (for example, uncertainty about the future); (4) quality of life (for example, losing freedom in life); (5) relationship and social participation (for example, experiencing a lack of understanding); and (6) daily functioning (for example, stopping working).

**Conclusion:**

Caregivers experience several health problems, even years after their relative's ICU episode. Healthcare providers should be focused not only on former ICU patients’ health, but also on their caregivers’, and need to signal and identify caregivers' health problems earlier in order to give them the appropriate care and support they need.

## How this fits in

Family caregivers of former ICU patients can develop physical, mental, social, and financial problems due to the ICU hospitalisation of their relative, which negatively affects their quality of life. As empirical research on this topic is scarce, knowledge of the nature and scope of this phenomenon is limited. This study gives a broad overview of experienced health problems of caregivers of former ICU patients, the consequences of these problem on their daily lives, and recommendations for health care providers to increase awareness for the problems found.

## Introduction

Family members can act as a crucial extension of the formal healthcare system. As 80% of adults requiring long-term care live at home or in community settings, family members provide 90% of this care.^1,2^ However, caregiving is associated with multiple types of burden for caregivers.^3^

This also applies to family caregivers of former ICU patients.^4–6^ Many patients who survive ICU hospitalisation deal with physical, cognitive, financial, and/or psychosocial problems years after ICU discharge. These symptoms are known as post-intensive care syndrome (PICS). But not only are former ICU patients at risk of adverse outcomes after ICU stay, their family caregivers can also develop physical, mental, social, and financial problems, owing to the ICU hospitalisation of their relative, known as PICS-F (post-intensive care syndrome, family).^4–6^ This burden on family caregivers appears to be present for a long time after ICU hospitalisation,^7^ and seems to play a major role in the life of former ICU patients’ family caregivers.

In a systematic review, psychosocial problems are reported most frequently, with prevalence rates varying between 25% and 50%. These include symptoms of anxiety, depression, and even post-traumatic stress,^4^ negatively impacting family caregivers' mental health-related quality of life (HRQL).^8^ Daily life interference is also high: half of the caregivers experience restrictions in practising hobbies and recreation.^4^ Half of the caregivers had to make adjustments to their working life to accommodate their caregiving responsibilities, and 14% of caregivers even had to stop working.^9,10^

In primary care, GPs have an identifying and coordinating role in the treatment of these symptoms and they have the opportunity to refer caregivers, for example, to a social worker or psychotherapist. Despite the symptoms found in previous studies, many GPs may not be familiar with the unique needs of these caregivers.^11,12^ While former ICU patients may turn to the ICU with their problems, family members’ problems will be presented to GPs first. Consequently, many caregivers probably miss the professional care they need. Therefore, there is an urgent need to thoroughly understand the consequences of ICU hospitalisation on family caregivers.^13,14^ As descriptive research into these problems is scarce, more insight is needed into the nature and impact of PICS-F, to inform relatives of former ICU patients and healthcare professionals. The aim of the present study was, therefore, to explore health problems in family caregivers of former ICU patients and the consequences in their daily life.

## Method

### Design & setting

An exploratory qualitative study with semi-structured interviews was conducted with caregivers of former ICU patients treated in a large academic hospital in the Netherlands. The interview study was carried out between April and May 2019. The consolidated criteria for reporting qualitative research (COREQ) guidelines for the design and analysis of this interview study were followed.^15^

### Participants

Family caregivers were defined as unpaid people who assist in the patient’s needs in daily care. Purposive sampling was used to include caregivers of patients treated between January 2016 and December 2018, who had been mechanically ventilated and admitted to the ICU for ≥5 days for a non-elective reason. These criteria were chosen as prior research found a mean ICU admission duration of 4.5 days.^16^ To pass the period of acute stress symptoms,^17^ interviews took place 3 months to 3 years after ICU discharge. Interview candidates had to be aged ≥18 years, and competent in the Dutch language. To avoid possible grief, caregivers of deceased patients were excluded. An ICU nurse selected a subsample of former ICU patients fulfilling the inclusion criteria from all past ICU admissions within the timeframe, via the electronic health record. Caregivers were iteratively recruited until data saturation was achieved. Subsequently, an information and invitation letter was sent to these patients. When informed consent was given by the patient, patients were asked to pass on a second letter of information regarding the background and aim of the study to a first-degree relative who had been close to them from the moment of ICU admission until the present. A first-degree family member was defined as, for example, a husband, a child, or a sibling. One caregiver was recruited by her GP.

### Data collection

Caregivers who wanted to participate could choose if the interview was located at their home or at the hospital. A trained interviewer (DS) conducted the interviews in the absence of the patient. In one interview, the patient was present in the background.

A semi-structured interview topic guide with open-ended questions was developed, pilot tested, and discussed with a mental health psychologist with experience in this research field. Huber and colleagues’ six dimensions of health were used as framework for the topic guide.^18^ During the interview process, few changes to the topic guide’s questions were made, based on experiences in the former interviews. Participants were asked to describe their experiences as openly as possible. The interview started with a brief summary of the patient’s ICU stay by the interviewer, followed by the question how the patient was doing now. The topic guide’s key questions are presented in Box 1, and the complete topic guide in Supplementary Box 1. Interviews were conducted until saturation was reached, meaning that no new information could be identified in the latest interview.

Box 1 **Topic guide’s key questions. The complete topic guide can be found in Supplementary Box 1. ICU = intensive care unit.**

**Table:** 

	***Sample interview questions***
*A* .	*Can you tell me how your family member is doing now?*
*B*.	*How did you experience the period of ICU admittance?*
*C*.	*Can you describe how you have experienced the period directly after ICU discharge?*
*D*.	*Compared to the period before ICU admission, do you feel different about yourself, mood-wise?*
*E*.	*Do you encounter other complaints since the ICU admission? For example, physical complaints*.
*F*.	*How do you encounter previously mentioned topics in daily life?*
*G*.	*Are there any topics we have not discussed yet, but could be relevant?*

Interviews were audio-taped and transcribed verbatim. Transcripts were sent back to give the participant the opportunity to make corrections or additions.

### Data analysis

Interviews were systematically analysed according to Braun and Clarke’s six-phases, including^1^ become familiar with the data,^2^ generate initial codes,^3^ search for themes,^4^ review themes,^5^ define themes, and^6^ write up.^19^ Findings from the interviews were categorised into six themes according to Huber *et al*’s six dimensions of health model^18^ (Box 2). The first two interviews were coded by three researchers (DS, MZ, and WG) independently. These data were discussed, replaced, or recoded until a consensus was reached. The remaining transcripts were coded by one researcher (DS) who also enumerated all the types of reported burden and identified representative quotes for the prominent subcategories. Remaining, inconclusive parts of the interviews were discussed with the other two researchers. The final codebook was reviewed and revised by the same three authors until consensus was reached. Coding was conducted by using ATLAS.ti (version 8.4.15).

Box 2 **Six dimensions of health indicators, covering 32 aspects of health.^18^ ADL = activities of daily living**

**Table:** 

**Bodily functions**	**Mental functions and perception**	**Spiritual or existential dimension**	**Quality of life**	**Social and societal participation**	**Daily functioning**
Medical facts	Cognitive functioning	Meaning or meaningfulness	Quality of life or wellbeing	Social and communicative skills	Basic ADL
Medical observations	Emotional state	Striving for aims or ideals	Experiencing happiness	Meaningful relationships	Instrumental ADL
Physical functioning	Esteem or self-respect	Future prospects	Enjoyment	Social contacts	Ability to work
Complaints and pain	Experiencing to be in charge or manageability	Acceptance	Perceived health	Experiencing to be accepted	Health literacy
Energy	Self-management		Flourishing	Community involvement	
	Resilience, sense of coherence		Zest for life	Meaningful work	
			Balance		

## Results

### Participants

**Figure 1.**
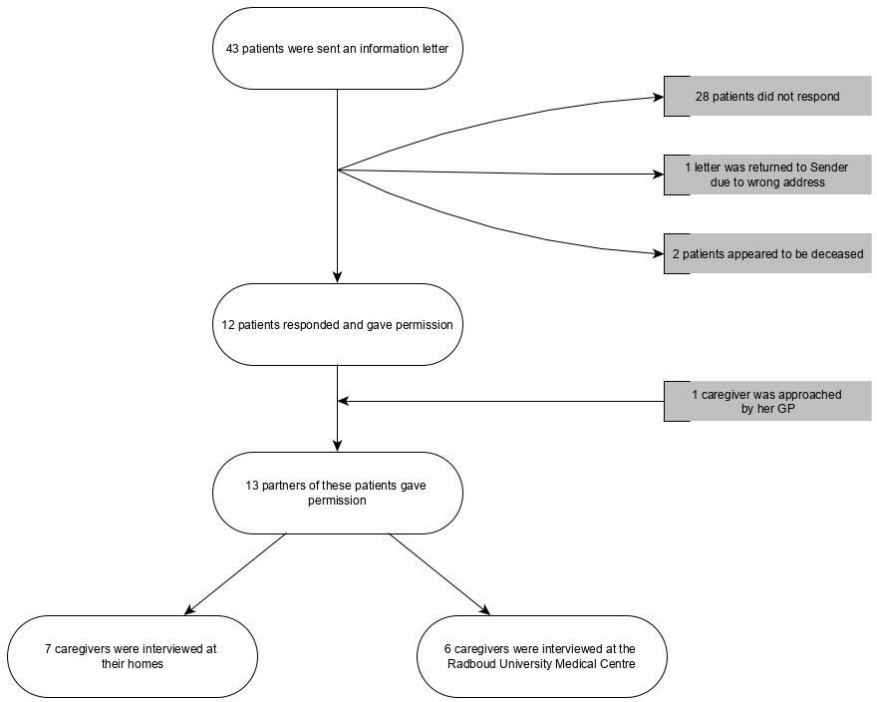
Flowchart: recruitment of participants

A subsample of 43 was selected and approached from a total of 271 former ICU patients. In total, 13 out of 43 (30% of) caregivers of former ICU patients were subsequently interviewed (Figure 1). All participants were partners of former ICU patients. Three were males, and the age ranged from 30–77 years. Except for two participants, all had children (children’s ages ranged from 8–51 years). Median time after ICU discharge was 10 months (minimum 3 months, maximum 3 years). Seven interviews took place at the caregiver’s home. Participant characteristics are shown in Table 1.

**Table 1.** Demographic characteristics of the participants

**Participant**	**Sex (M/F**)	**Age, years**	**Children, *n***	**Diagnosis of patient**	**Time since discharge from ICU**
A	F	59	2	Ruptured aortic aneurysm	1 year 3 months
B	F	30	None	OHCA	9 months
C	F	63	1	Respiratory failure	10 months
D	M	61	None	Respiratory failure	8 months
E	F	58	2	OHCA	8 months
F	F	55	2	Respiratory failure	3 months
G	F	50	3	OHCA	1 year 5 months
H	M	55	3	Respiratory failure	1 year 7 months
I	F	56	2	Respiratory failure	10 months
J	F	47	2	Polytrauma	9 months
K	F	73	2	Cardial ischaemia	1 year 10 months
L	M	77	3	Aortic dissection	3 years
M	F	46	2	Subarachnoid haemorrhage	1 year 5 months

OHCA = out of hospital cardiac arrest

### Themes

In total, 21 sub-themes were derived from the interviews and categorised into the six themes according to Huber *et al*’s six dimensions of health model (Box 3).^18^

Box 3 **Themes exemplifying caregivers' health problems according to Huber *et al*’s six dimensions of health.^18^**

**Table:** 

**Theme**	**Sub-theme**	**Example of found problem**	**Representative quote**
**Physical health**	Energy	Tiredness	*'I’m very caring. I like to care. But sometimes it comes at the expense of myself and that expresses itself as tiredness.'* (D)
		Sleep problems	*'I don’t sleep instantly. First, I have to be sure he* [the patient] *is sleeping. Then I dare to take a nap. Usually that’s not until the morning.'* (C)
	Complaints and pain	Neck pain	*'Stress! Tenderness! It started to accumulate in my neck and shoulders. Therefore I received physiotherapy for, I think, three months. It crept in without me being aware…'* (B)
		Headache	*'At some point, I got a sort of headache and neck pain...'* (G)
		Feeling sick more often	*'In two months I’ve been sick four times, like a sort of flu, with a terrible headache, nausea and vomiting, which I usually never have. I never have headache. I just didn’t understand, until I realised: maybe it’s a manifestation of…'* (A)
		Chest pain	*'One time, I went to the GP because I had chest pain, within the heart region. I thought: It’d be better to have it checked before I collapse as well.'* (M)
	Medical observations	Weight changes	*'At the moment I weigh 63.8 kilograms. Last year I weighted 76. So that’s 13 kilograms less.'* (C)
**Mental health**	Cognitive functioning	Amnesia	*'During the holidays I bought the kids tickets for the theatre. Great show. Very expensive tickets. And I just forgot. With all the hustle … The children were very sad and upset. I felt really bad about it.'* (M)
	Emotional state	Difficulty expressing emotions	*'When he* [the patient] *is very sad, it makes me sad as well. Then I want to cry, but I block. That’s because I’m afraid to start crying and won’t be able to stop anymore … Since there’s so much which still needs to be processed.'* (I)
	Stress	More stress	*'Just restlessness. 24 hours of restlessness.'* (E)
	Depressive feelings	More depressive feelings	*'Cleaning the house. I don’t feel anything for it anymore. I don’t care anymore. I take one day at a time, or I don’t take any.'* (C)
	Anxiety	Fear of recurrence	*'If she* [the patient] *is in the bathroom a little long, I go upstairs to touch base. It just has become automatic. For all we know, she lies there as I experienced before.'* (L)
	Self-esteem and manageability	Shame	*'I don’t like the way I behave this way* [considering the patient’s body as scary]*. I don’t blame him, but I blame myself…'* (J)
**Existential dimension and future**	Spiritual dimension and meaning	Changed spirituality	*'Occasionally a little prayer and a Hail Mary really supported me.'* (H)
	Acceptance	Difficulty accepting the situation	*'Accept it the way it is. It made the situation more bearable and easier, I think. Well, it wasn’t easy, but because of that we managed to get through this.'* (M)
	Future prospects	Uncertainties in the future	*'When comes the quality time we hope to get some day?'* (J)
**Quality of life**		Impaired freedom	*'We lived very relaxed, everything was possible. Which is delightful if everything goes well, but has turned over completely now. However, it’s not necessarily a bad one. Really.'* (A)
**Relationship and social participation**	Relationship	Impaired intimacy	*'Well, you don’t actually have any physical contact, not to mention sex.'* (J)
	Social participation	Losing friends	*'Now you realise who really is close to you. It takes a while to accept, but you don’t actually realise it until now.'* (D)
**Daily life**	Hobbies and leisure	Recreational limitations	*'Taking a nice walk. In Berlin we walked about fifteen kilometres a day. Lovely, isn’t it? You’ll see everything. Well, that’s never going to happen anymore.'* (A)
	Family and home	More responsibilities at home	*'Look, my husband has become sick and I haven’t, but I’m involved just as much.'* (I)
	Work and financial matters	Financial uncertainties	*'Financially, we don’t dare to spend a lot of money yet. So sports club I don’t dare yet and swimming neither actually. Soon I can swim in the pond over here, that’s free.'* (M) [Owing to M’s patient, who is currently depending on benefit]

#### Physical health

Interviewees expressed feeling more tired, which was most often worst shortly after hospital discharge. Having obligations at their job and giving care to their partner simultaneously during a period of emotional hard times was frequently expressed as a cause of tiredness. One participant reported excessive weight loss owing to a lack of appetite:

*‘I didn’t want to eat, I didn’t want to live. I thought: if he’s gone, I have to be gone.’* (C)

Furthermore, tightening neck pain, headache, feeling sick more often, weight changes, and episodes of chest pain were reported.

Caregivers also mentioned tiredness as a consequence of poor sleep owing to feelings of stress and concerns about the patient. Some had hospital-related nightmares and re-experiences shortly after hospital discharge, disturbing their sleep.

#### Mental health

##### Cognitive functioning

Several participants mentioned increased amnesia, in particular with regard to remembering names, appointments, and things they had to do. Furthermore, caregivers reported being distracted more easily and having problems ‘keeping their heads in the game’ with simple tasks.

##### Emotional state

Caregivers reported recurrent feelings of anger and sadness because life was not going the way they wanted. They also described sadness due to difficulty dealing with uncertainties in the future. Moreover, caregivers described feeling like their head was exploding with emotions, and that they could not handle any more. Caregivers seem to have difficulties expressing these emotions, as they put aside their own emotions. They perceive their life issues inferior to those of the patient, for example:

*‘I’m so busy, sometimes I think: I can’t keep going, I’m stuck. Shall I tell him* [the patient]*? He probably has something else on his mind.’ (A*)

##### Stress

Caregivers reported stress owing to frequently re-experiencing ICU-related phenomena. Described triggers for this were: talking about it, the sound of an ambulance siren, and even hearing the patient’s voice calling. Two participants successfully underwent eye movement desensitisation and reprocessing (EMDR) therapy, reporting that they encountered fewer re-experiences. A higher state of alertness towards the patient was also mentioned frequently. Especially when awake at night, caregivers wanted to make sure the patient was still breathing. Another cause of stress was the feeling of having to keep too many plates spinning besides the informal care of the patient; for example, taking care of their children and parents, work, and their own health issues.

Caregivers stated they even experienced better mental health *during* ICU hospitalisation than they did before. These participants explained that they were already overloaded as informal caregivers of their chronically ill partner prior to hospitalisation; ICU admission offered them more time and space for themselves instead of having to take care of their partner continuously.

##### Depressive feelings

Some caregivers mentioned being more gloomy and negative about things. They did not feel like doing anything they used to like doing, for example:

*‘Being negative about everything. Passing judgements on everything, everything was foolish …’* (E)

##### ​Anxiety

During the ICU hospitalisation, caregivers had anxious and challenging feelings about the ICU setting, and thought their partner was going to die. Caregivers also described fear of having to go on alone. Consequently, they were afraid of recurrence of the ICU diagnosis and the patient’s health in the future. On the other hand, caregivers also feared leaving the patient alone if they would pass away themselves, as the patient was totally dependent:

‘*Fear that I pass away, but the real fear is: how will my wife save herself then? As a matter of fact, she can’t miss* [function without] *me.’* (L)

#### Self-esteem and manageability

As caregivers state they need to lead during the process of ICU hospitalisation, the never-ending feeling of powerlessness despite their efforts is frustrating. Not only while waiting for a scan’s result, but also some felt like they could not go on with their lives as they were effectively ‘bonded’ to treatments aking place for years.

#### Existential dimension and future

##### Spiritual dimension and meaning

After the ICU hospitalisation, several caregivers started to think differently about what was really important in their lives, for example:

*‘No matter how beautiful we live in this lovely thatched farmhouse with a marvellous swimming pool, it is not worth it to me.’* (H)

Interviewees experienced support from their religion or people of their church. For example, one man even stated:

*‘I didn’t have anything to do with the church. However, I’ve been thinking differently about this. It helped me in a positive way.’* (D)

##### ​Acceptance

Although most participants said they had accepted their current situation, for some it appeared to be difficult to accept. Some described feelings of disbelief and unfairness, for example:

‘*If it was just caused by ageing, so be it. But we both did our best to live healthy our whole lives, and then you get this! This mess.’* (L)

##### Future prospects

Many caregivers reported uncertainty about the future with their former ICU patient partner. Fear of deterioration of their partner’s health, financial matters, housing, and holidays abroad: people did not know where they would stand in a few years and what of these things would still be possible. Therefore, caregivers started thinking about their future more consciously as they realised life is finite:

*‘How long do you have left and what do you want?’* (E)

#### Quality of life

Many caregivers stated that the ICU hospitalisation brought them down to earth and made them appreciate the small things in life more, which actually enriched their lives:

*‘Maybe it* [my life] *has gained another dimension, making it better.’* (G)

On the other hand, some caregivers had to give up the flexibility and freedom of their former life, for example:

*‘You’re a bit around the house, because they* [home carer] *can show up at any moment to take a look at his wound, which has to be taken care of.’ (I*)

#### Relationship and social participation

##### ​Relationship

Several caregivers described the loss of a conversation partner with whom to unload their emotional baggage, as their life companion was critically ill. Later on, as some caregivers had to deal with their own health problems, they sometimes felt a lack of support and attention for their own situation from the patient because he or she was too busy with his or her own problems and rehabilitation. Despite the limited memory of their ICU stay, patients appear to be left with a different experience of the ICU hospitalisation than their caregivers. Therefore, several caregivers experience differences in visions and the way they both deal with the patient’s problems and rehabilitation, for example:

*‘Since I have lost my job, I was very sick myself. I’m very angry he* [the patient] *started cycling more and more to get rid of his own stress, but because of that, there was no time for me. I have to deal with my own issues at home.’ (J*)

Intimacy and sexuality was also changed after ICU hospitalisation. Caregiver J described the patient’s impaired shoulder functions after his accident: ‘*For one year, I haven’t had any arm around my shoulder…’.* She stated her partner’s body changed so much after ICU hospitalisation, she found it scary and was ashamed of herself reacting towards him this way.

Emotional impact on the caregivers' children was also noticed, which was hard to face and difficult to deal with. Caregivers were worried whether or not they had chosen the right approach in order to offer them guidance on the situation, for example:

‘*I think one of the hardest things to do is informing the children. What would be the best way to do so? Yet, I have to deal with a son of seventeen, a daughter of twenty-two, and a son of twenty-five years old, but I still have to tell them something’s going terribly wrong.’* (H)

##### Social participation

Caregivers reported they had lost friends. Furthermore, more distant contacts seem to downplay the situation or show interest to only a very superficial level. On the other hand, during ICU hospitalisation, intense interest of other distant or virtually unknown people was considered exasperating and time-consuming, for example:

*‘After this happened to my husband, I had a fight with a lot of people, as I thought they interfered with things they shouldn’t interfere with.’* (E)

As several patients were already chronically ill before ICU admission, their caregivers used to encounter a lack of understanding and compassion from social contacts and at work. Caregiver F cited a colleague:

*‘Oh, he’s on the ICU? Oh, then there must be *something serious* going on …’*

#### Daily functioning

##### Hobbies and leisure

Most of the caregivers experienced limitations in practising their hobbies and recreation together, mostly owing to the patient’s physical limitations and uncertainties in the future. When going out together, patients were completely dependent on their caregivers, for example:

*‘Sometimes it just looks like I’m the mother and he’s the kid …’* (F)

Moreover, some noticed they spent less time on their individual leisure or sports, which moved to the background as they were more busy finding practical solutions for the patient’s problems, or felt bad that the patient would be housebound and stayed at home to accompany him or her. Some caregivers were called home by their partners because something was wrong, or they were continuously preoccupied with their thoughts during their leisure time.

##### ​Family and home

Caregivers reported having a lot more daily caregiving tasks than they did before. They had, for example, saddled themselves with household tasks the patient used to do:

*‘At once, aside from your father’s duties, you have mother’s duties as well.’* (H)

Besides taking care of their partner and children, some had to give informal care to their parents and work simultaneously.

##### ​Work and financial matters

Shortly after ICU hospitalisation, several caregivers experienced extreme pressure to continue their jobs. They felt like employers did not assess their situation properly and played it down easily, for example:

‘*Nowadays, people seem to think: as long as you come to work … Because you have to keep in touch with your job … But it doesn’t work like that if you’re tired.’* (F)

Even after a long period of hospitalisation, several caregivers started to work less, quit their job, or had to sell their business because they just could not handle it anymore or wanted to spend more time with their families.

Although none reported serious financial problems, financial uncertainty for the future appeared to be a concern for many. Two reported having sold their houses in order to downsize. One caregiver couldn’t afford to buy his wife a custom bike.

## Discussion

### Summary

This exploratory study shows that family caregivers of ICU survivors experience various health problems even years after ICU discharge, including physical problems such as tiredness, as well as psychosocial problems. Caregivers described cognitive complaints owing to anxiety, sleep problems, and recurrent negative feelings and emotions which, in turn, appear to be difficult to express. Furthermore, the anxiety of having to go on alone, feelings of stress, a higher state of alertness, and having to deal with uncertainties in future was experienced as extremely inconvenient, as caregivers desperately desire to ‘paddle their own canoe’. Additionally, caregivers experience a lack of support for themselves from their partner, as well as from social contacts and at work. Therefore, caregivers are forced to work less and have to deal with financial uncertainties. The impact on their daily life goes far beyond work, as caregivers also have to give up leisure activities as their responsibility within the family increases. Healthcare providers need to become aware of previously described symptoms to give these relatives the support and care they need.

### Strengths and limitations

A methodological strength of this study is the patient’s absence during the interview (except one being present at the background), so he or she could not influence their caregiver’s free speech. In addition, face-to-face interviews are useful to explore sensitive topics in depth. Another strength was the opportunity given to the caregivers to revise the transcripts afterwards, which only one person did. By selecting caregivers of patients with a large variation in diagnosis, a breadth of perspectives were included, increasing the generalisability of the research.

This study has several limitations that need to be addressed. The study aimed to obtain a diverse, purposively sampled group of caregivers, and it aimed to select multiple types of family members, but only partners of patients responded. It would be worth knowing what problems are encountered by, for example, children of patients. It is also important to bear in mind the possible selection bias in these responses, as only a small proportion of the people approached responded. Furthermore, only a single-centre ICU was used, so caregiver’s experiences could be determined by this centre’s care. However, the results are in line with previous research.^4^ In addition, six interviews took place within the hospital, which could have led to some caregivers not feeling comfortable. Caregivers were, however, given the choice in advance of where the interview would take place so, in these cases, it was the caregiver’s preference to be interviewed at the hospital.

### Comparison with existing literature

Symptoms of psychosocial burden were also found in previous studies.^4^ The impact on daily life found in the present study is in line with previous quantitative research, which also reported reductions in employment and restrictions in practising hobbies.^4,9,10,20^ Although these were previously described in US studies, the interviewed partners did not experience serious financial problems, such as difficulties paying for basic needs.^21,22^ This could be owing to the Dutch social security system, which likely offers more protection against financial setbacks. Added to that, every person who lives or works in the Netherlands is legally obliged to take out standard health insurance to cover the cost of, for example, consulting a GP, hospital treatment, and prescription medication.

Descriptive research on these topics is scarce. Some qualitative studies reported symptoms of sleep disorders, sadness, difficulties explaining the situation to the children, and a sense of distance in caregivers' relationships, which is in line with the present study's findings.^23,24^ The observed differences in coping by the caregiver and ICU patient could be owing to different experiences during the ICU hospitalisation, as was previously suggested by Young *et al*, who compared mental health symptoms of ICU and elective cardiac surgery patients and their relatives.^25^ To the authors' knowledge, the lack of support caregivers experience from their partners has not been described previously. A contributing factor for this phenomenon could be the chronic illnesses the caregivers experienced themselves. Two caregivers reported having cancer, and two caregivers suffered from chronic back pain. Furthermore, to the authors' knowledge, the impact on caregivers' physical health and cognitive functioning has not been described previously either.

Reported symptoms of family caregivers are partly in line with symptoms former ICU patients have to deal with. Both caregivers and patients experience not only mental symptoms — for example, depressive feelings, cognitive impairment and lack of control of their lives — but also physical symptoms such as sleeplessness, fatigue, and listlessness.^26^

### Implications for research and practice

While this exploratory study sheds light on the nature of the health problems that caregivers of ICU patients encounter, there are still many unanswered questions about the scope of these problems. In order to give caregivers appropriate and timely support, healthcare professionals should aim to identify caregivers' health problems early. More knowledge and awareness about health problems of caregivers of former ICU patients is needed. Together with ICU staff, GPs have ongoing opportunities to signal, monitor, and manage the health of both former ICU patients and their caregivers following ICU discharge.^27–29^

Prior studies have noted the importance of thoroughly understanding the consequences of ICU hospitalisation on caregivers,^13,14^ and the present study shows these problems and their consequences in daily life. GPs should obtain a comprehensive picture of caregiving stress and estimate the impact on the wellbeing of the caregiver.^30^ However, psychosocial items concerning patient and caregiver appear to be less desirable topics during anamnesis.^31^ Previously developed guidelines for general caregiver assessment could help GPs to systematically gather this information.^3,32^ However, in order to assess specific post-ICU health problems in caregivers and develop targeted interventions, measures of caregivers outcomes that are responsive to intervention need to be identified.

When healthcare providers obtain a clear understanding of the burdens caregivers encounter, caregivers may benefit from family-based models of care,^33,34^ along with cognitive therapy and increased social support.^35^ As families of ICU patients may have different needs, the provision of individualised, family-centred care is likely to have a positive influence.^34^ Furthermore, distressed family members may benefit from early cognitive behavioural therapy, as trauma survivors with acute stress disorder reported less intense symptoms after therapy.^36,37^ Peer support programmes for caregivers have also been suggested, in order to give individuals the opportunity to normalise their feelings and reduce any feelings of social isolation that they may be experiencing.^38,39^

However, the amount of interventions and studies to reduce or mitigate consequences of the caregivers burden are limited. Despite these promising approaches, more research into these interventions is needed. As the Dutch healthcare system and ICUs appear to be similar to those of other Western countries, the results of this study can be used to further develop interventions to assess and prevent health problems in caregivers in primary care.^40,41^

In conclusion, caregivers of partners hospitalised in the ICU experience several physical and psychosocial problems years after ICU discharge of their loved one, which has a major impact on their daily life. Healthcare professionals should take into consideration that a single ICU hospitalisation offers them two people in need of their care: patients suffer from residual symptoms owing to critical illness and ICU hospitalisation, but their caregivers also encounter health problems as a consequence of this stressful event. Healthcare providers need to become aware of mental and physical complaints in order to give these relatives the care they need.
